# 15215 Female sex worker experiences with intimate partner violence screening by health care providers

**DOI:** 10.1017/cts.2021.742

**Published:** 2021-03-30

**Authors:** Jessica L. Zemlak, Kamila A. Alexander, Deborah H. Wilson, Susan G. Sherman

**Affiliations:** 1 Johns Hopkins School of Nursing; 2 Johns Hopkins School of Public Health

## Abstract

ABSTRACT IMPACT: This work will inform the need for more trauma-informed approaches to violence screenings among marginalized populations by health care providers. OBJECTIVES/GOALS: Female sex workers (FSW) experience high rates of intimate partner violence (IPV) which may have negative reproductive health consequences. Routine IPV screening by healthcare providers (HCP) is recommended. This study examines how FSW experience IPV assessments by HCP. METHODS/STUDY POPULATION: This qualitative descriptive study is nested within EMERALD, a longitudinal cohort study of street-based FSW. EMERALD assesses a structural community-level intervention on HIV and STI risks among FSW. Participants were recruited for EMERALD using time-location sampling to identify a sampling frame of venues and times where sex work is likely to occur. Inclusion criteria for this qualitative study include: participating in EMERALD, age 18-49, and willingness to participate in one phone interview. Twenty-two semi-structured individual qualitative interviews were conducted. Two coders, using thematic analysis, applied deductive codes and inductive coding to identify themes. RESULTS/ANTICIPATED RESULTS: Three themes emerged from participant interviews regarding IPV screening. Inconsistent screening practices: Despite frequent reproductive health visits among participants, many women did not recall IPV screening by a HCP. Stigma as a barrier to disclosure: Women described feeling judged by HCP regarding their frequency of visits for reproductive concerns, sex work, and substance use and did not trust disclosing violence to HCP. Transactional health encounters: During visits, HCP were focused on addressing women’s immediate concerns; if the HCP did ask about IPV women felt the questions were asked part of a required protocol and not driven by a concern to deeply understand their lives. DISCUSSION/SIGNIFICANCE OF FINDINGS: FSW described inconsistent IPV screening practices by HPC. Participants expressed feeling stigmatized by HCP and that encounters with HCP did not foster trust for women to disclose IPV experiences. There is a need for HPC training in universal IPV screening focused on relationship and trust building to facilitate disclosure.

